# Free-breathing three-dimensional whole-heart adiabatic T1ρ mapping for non-contrast tissue characterization at 0.55T

**DOI:** 10.1016/j.jocmr.2025.102676

**Published:** 2025-12-24

**Authors:** Dongyue Si, Michael G. Crabb, Simon J. Littlewood, Karl P. Kunze, Claudia Prieto, René M. Botnar

**Affiliations:** a School of Biomedical Engineering and Imaging Sciences, King's College London, London, United Kingdom; b MR Research Collaborations, Siemens Healthcare Limited, Camberley, United Kingdom; c School of Engineering, Pontificia Universidad Católica de Chile, Santiago, Chile; d Millennium Institute for Intelligent Healthcare Engineering, Santiago, Chile; e Institute for Biological and Medical Engineering, Pontificia Universidad Católica de Chile, Santiago, Chile; f Institute for Advanced Study, Technical University of Munich, Garching, Germany

**Keywords:** 3D, Low-field MRI, T1ρ mapping, Myocardial tissue characterization, Adiabatic spin-lock pulses

## Abstract

**Background:**

Commercial 0.55T low-field magnetic resonance imaging (MRI) systems have recently become available, offering the potential to enhance global accessibility to MRI. T1ρ mapping is an emerging quantitative cardiac MR imaging technique capable of detecting myocardial disease without the need for contrast administration. However, experience with cardiac T1ρ mapping at low-field strength remains limited. This study aims to develop and validate an efficient, free-breathing three-dimensional (3D) high-resolution adiabatic T1ρ mapping sequence for non-contrast whole-heart tissue characterization at 0.55T.

**Methods:**

The proposed 3D T1ρ mapping research sequence acquires four interleaved volumes with different contrast weightings using saturation and adiabatic spin-lock preparation pulses, and a 3-parameter fitting method is used to calculate T1ρ maps. Two-dimensional (2D) image navigators are acquired for non-rigid motion-compensated image reconstruction, enabling 100% respiratory scan efficiency. Phantom and in-vivo experiments in 10 healthy volunteers were conducted to evaluate the accuracy and precision of the proposed 3D sequence in comparison with 2D T1ρ mapping sequences.

**Results:**

Phantom T1ρ values measured using the proposed 3D sequence showed strong agreement with the 2D reference (R^2^ = 0.997), demonstrating high accuracy and reduced sensitivity to heart rate variations. In-vivo experiments in healthy subjects demonstrated that the proposed sequence is feasible for acquiring whole-heart T1ρ maps with 2 mm isotropic resolution in an efficient scan time of 6.6 ± 0.5 min. The mean myocardial T1ρ value obtained with the 3D sequence was slightly higher than that of a conventional 2D breath-hold sequence (112.8 ± 16.7 vs. 106.1 ± 15.1%, p<0.01), while coefficient of variation (CV) was slightly lower (10.2 ± 5.2 vs. 11.4 ± 4.4%, p = 0.02).

**Conclusion:**

The proposed sequence enables 3D free-breathing high-resolution adiabatic T1ρ mapping and shows promising potential for non-contrast whole-heart tissue characterization at 0.55T.

## Graphical abstract

**Figure:**
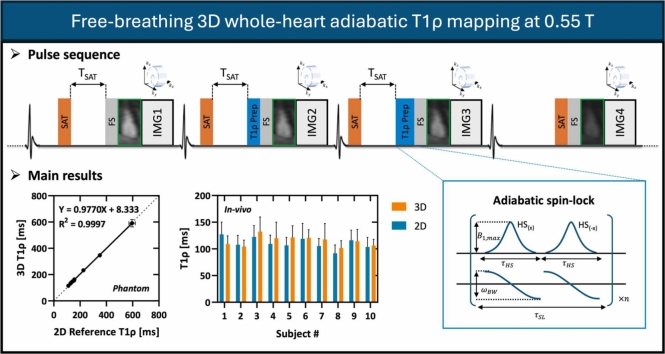

## 1. Background

Cardiovascular magnetic resonance (CMR) is an established non-invasive and radiation-free imaging modality for the assessment of a wide range of cardiac diseases. However, the widespread clinical adoption of CMR remains limited due to the high costs and restricted accessibility especially in less developed regions [1]. More recently, commercial low-field magnetic resonance imaging (MRI) systems (<1T) have become available, which may improve accessibility to MRI across the world because of the lower costs compared to 1.5T and 3T systems [2].

Cardiac T1ρ mapping is an emerging CMR quantitative parametric imaging technique that can detect myocardial disease without the use of contrast agent [3]. Usually, T1ρ mapping is implemented with continuous wave (CW) spin-lock (SL) pulses at routine field strengths of 1.5T or 3T [3]. However, CW-SL is restricted to a low frequency of 175 Hz because of the limited maximum power of the RF amplifier on a commercial 0.55T system [4]. As an alternative, adiabatic-SL preparation pulse was also proposed and applied for T1ρ mapping at 0.55T [4], [5], [6], which achieved a higher maximum frequency of 500 Hz due to the reduced peak RF power requirements compared to CW-SL [4]. Furthermore, adiabatic T1ρ mapping resulted in better precision compared to CW T1ρ mapping as it is less sensitive to field inhomogeneities [5], [6]. However, a 2D breath-hold acquisition protocol was used in the previous study, limiting whole-heart imaging [4]. To address the above limitations, we propose a more time-efficient and patient-friendly free-breathing high-resolution 3D adiabatic T1ρ mapping sequence that can be performed on a commercial 0.55T MRI system.

## 2. Methods

### 2.1. Sequence design

**Fig. 1:**
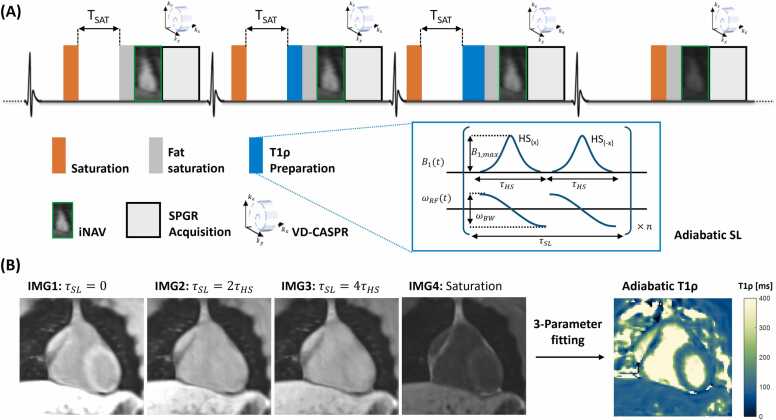
Framework of the proposed 3D adiabatic T1ρ mapping research sequence. **(A)** Pulse sequence diagram. T1ρ preparation is performed with adiabatic SL that consists of multiple pairs of HS pulses. Four interleaved 3D volumes are acquired in repeated cycles of four heartbeats with electrocardiogram triggered SPGR acquisition. The first three volumes (IMG1-IMG3) are prepared with saturation pulse with the same recovery time (T_SAT_), while the last volume (IMG4) is prepared with saturation pulse without delay time. IMG2 and IMG3 are also prepared with adiabatic-SL consisting of 2 and 4 HS pulses with SL duration ($\tau_{\mathrm{SL}}$) of $2\tau_{\mathrm{HS}}$ and $4\tau_{\mathrm{HS}}$, respectively. 2D iNAVs are performed before data acquisition in each heartbeat, and a variable-density Cartesian trajectory with spiral-like profile order (VD-CASPR) is adopted with 4-fold under-sampling. **(B)** T1ρ map calculation. The four volumes with different contrast weighting (IMG1-IMG4) are used to calculate a 3D T1ρ map voxel-by-voxel using a 3-parameter fitting method. *SL* spin-lock, *2D* two-dimensional, *3D* three-dimensional, *HS* hyperbolic-secant, *SPGR* spoiled gradient echo

The sequence framework of the proposed 3D adiabatic T1ρ mapping is illustrated in Fig. 1. T1ρ preparation is performed with adiabatic-SL which consists of multiple pairs of adiabatic full passage hyperbolic-secant (HS) pulses with opposite phases. The HS pulse has a duration of $\tau_{\mathrm{HS}}$, and is defined by the following amplitude ($B_{1}$) and frequency ($\omega_{\mathrm{RF}}$) modulation functions:

$$B_{1}\left(t\right)=B_{1,\max}\mathrm{sech}\left(\beta t\right)$$

$$\omega_{\mathrm{RF}}\left(t\right)-\omega_{0}\;=-\mu \beta \tanh\left(\beta t\right)$$

where $t\in [-\frac{\tau_{\mathrm{HS}}}{2},\frac{\tau_{\mathrm{HS}}}{2}]$, $B_{1,\max}$ is the peak $B_{1}$ amplitude, β is a truncation factor characterizing the width of the pulse bell, $\;\omega_{0}$ is the Larmor frequency, and $\omega_{\mathrm{BW}}=2\mu \beta$ defines the bandwidth of $\omega_{\mathrm{RF}}\left(t\right)$. In this study, $\tau_{\mathrm{HS}}$ = 15 ms, $B_{1,\max}$ = 11.7 µT, $\omega_{\mathrm{BW}}$ = 800 Hz, and μ= 4 were used for the HS pulse, which were optimized and demonstrated a good performance at 0.55T according to our previous study [4].

For T1ρ mapping, four interleaved 3D volumes are acquired in four heartbeats with an electrocardiogram (ECG) triggered spoiled gradient echo readout (Fig. 1**A**). The first three volumes are prepared with saturation (SAT) pulses with the same recovery time (T_SAT_), which is the maximal achievable delay time within one cardiac cycle for a given trigger delay. While the last volume is prepared with a SAT pulse without delay time. The second and third volumes are also prepared with adiabatic-SL pulses consisting of 2 and 4 HS pulses with SL duration ($\tau_{\mathrm{SL}}$) of $2\tau_{\mathrm{HS}}$ and $4\tau_{\mathrm{HS}}$, respectively. 2D image navigators (iNAVs) are performed before data acquisition in each heartbeat to detect respiratory motion in left-right and foot-head directions [7]. As a fat saturation pulse (flip angle = 180°) is performed immediately before the iNAV, only six iNAV lines were acquired to minimize the recovery of the longitudinal magnetization of fat and to consequently improve the efficiency of fat suppression at low-field strength, as shown in a previous study [8]. A variable-density Cartesian trajectory with spiral-like profile order (VD-CASPR) is adopted with four-fold under-sampling [9]. The acquired data are reconstructed with inline non-rigid motion corrected iterative-SENSE and offline high-dimensional patch-based low-rank regularization (HD-PROST) [10]. In this study, the HD-PROST regularization parameter (λ) was empirically set to 0.05 according to limited parameter search [10]. The 3D T1ρ map is then calculated offline from the four volumes with different contrast weighting voxel-by-voxel using a 3-parameter fitting method (Fig. 1**B**) [11].

### 2.2. MR experiments

Phantom experiments were performed with a standard 1.5T T1MES phantom [12]. Reference T1ρ values were measured with a fully sampled single echo gradient echo sequence, using TR of 10 s to ensure sufficient recovery of the longitudinal magnetization after every readout. First, to evaluate the accuracy and precision of the proposed 3D T1ρ mapping research sequence, phantom acquisition was performed with a simulated heart rate of 60 bpm and T_SAT_ of 600 ms. Second, to test the performance at different heart rates, the proposed sequence was performed at different simulated heart rates ranging from 40 to 120 bpm (R-R interval from 500 ms to 1200 ms), leading to a T_SAT_ ranging from 300 to 1100 ms.

In-vivo experiments were performed on 10 healthy volunteers (7 males, 30 ± 4 years) to validate the accuracy and precision of the proposed 3D T1ρ mapping sequence in comparison with a 2D breath-hold T1ρ mapping research sequence.

All MR experiments were performed on a clinical 0.55T MR scanner (MAGNETOM Free.Max; Siemens Healthineers, Erlangen, Germany) using a 6-channel chest array and a 9-channel embedded posterior receiver coil. ECG trigger signal was recorded from an external ECG monitoring system (Expression MR400, Philips Healthcare, Best, The Netherlands). The in-vivo experiments were approved by the local institutional review board (REMAS 8700). Written informed consent was obtained from all subjects before imaging. Image processing and data analysis were implemented in MATLAB R2023a (MathWorks, Natick, Massachusetts). Sequence parameters in this study are summarized in **Table S1**. Data analysis methods are detailed in supplemental files.

## 3. Results

### 3.1. Phantom results

**Fig. 2:**
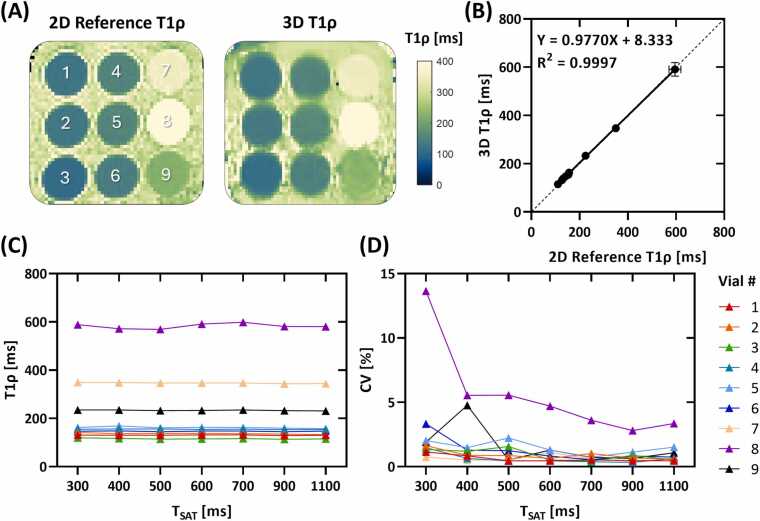
Phantom results. **(A)** Phantom T1ρ maps acquired with 2D fully sampled reference sequence and the proposed 3D T1ρ mapping sequence with simulated heart rate of 60 bpm and saturation recovery time (T_SAT_) of 600 ms. **(B)** Linear regression plot of phantom T1ρ measured by 3D T1ρ mapping in comparison with 2D reference values. **(C, D)** Phantom T1ρ mean value and CV measured by 3D T1ρ mapping with T_SAT_ ranging from 300 to 1100 ms and corresponding simulated heart rates of 120 to 40 bpm. Phantom T1ρ mean values indicate negligible sensitivity to heart rate especially for phantom vials that mimic myocardium (vial #1–6). T1ρ CVs were generally elevated with shorter T_SAT_ delays at higher heart rates, primarily due to reduced recovery of longitudinal magnetization. This effect is more pronounced for phantom vials with longer T1 and T1ρ values that mimic the blood pool (vial #8, 9). However, CVs were less sensitive to heart rate variations in phantom vials mimicking myocardium, remaining below 4% across all T_SAT_ values. *CV* coefficient of variation, *2D* two-dimensional, *3D* three-dimensional

Phantom T1ρ maps acquired with the 2D fully sampled reference sequence and the proposed 3D T1ρ mapping sequence with T_SAT_ of 600 ms are shown in Fig. 2**A**. 3D T1ρ mapping showed strong agreement with reference T1ρ values measured in the phantom, with a linear correlation of y = 0.9770x + 8.333 and R^2^ = 0.997 (Fig. 2**B**). The heart rate sensitivity of the proposed 3D T1ρ mapping sequence is shown in Fig. 2**C, D**. Both T1ρ mean values and coefficients of variation (CVs) measured with the proposed sequence indicate negligible sensitivity to heart rate for phantom vials that mimic myocardium (vial #1–6).

### 3.2. Healthy subjects

All 10 healthy subjects successfully completed the in-vivo experiments. The proposed sequence was acquired within a scan time of 6.6 ± 0.5 min with an average heart rate of 60 ± 5 bpm. T_SAT_ time was in the range of 530 to 740 ms for all subjects.

**Fig. 3:**
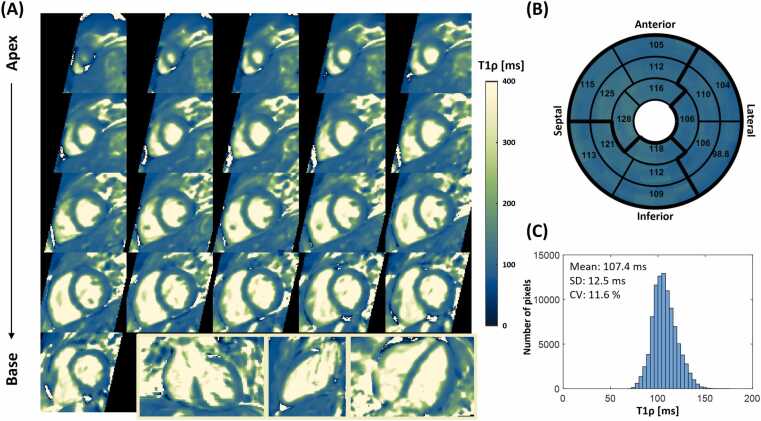
Representative T1ρ maps of a healthy volunteer measured by the proposed 3D T1ρ mapping sequence. **(A)** 3D T1ρ maps in different orientations including short-axis views from apex to base, coronal view, 2-chamber, and 4-chamber view. **(B)** Bull's-eye plots of whole-heart T1ρ. The mean T1ρ values in the 16 American Heart Association segments are presented. **(C)** Histogram of the myocardial T1ρ over the whole left ventricle. The T1ρ mean, SD and CV across the whole left ventricle are shown. *SD* standard deviation, *CV* coefficient of variation, *3D* three-dimensional

3D whole-heart T1ρ maps and bull’s-eye plot of a representative healthy volunteer are shown in Fig. 3. Myocardial T1ρ maps acquired with the proposed 3D sequence exhibited high spatial uniformity, with an average T1ρ of 107.4 ± 12.5 ms across the entire left ventricle, corresponding to a CV of 11.6% (Fig. 3**C**).

**Fig. 4:**
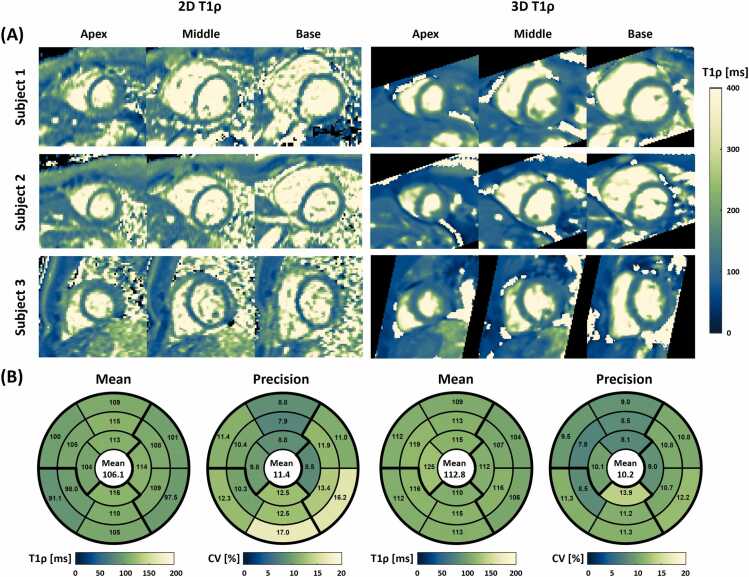
Results of in-vivo experiments. **(A)** Representative short-axis T1ρ maps of three healthy subjects obtained with the proposed 3D T1ρ mapping sequence and a breath-hold 2D T1ρ mapping sequence. 3D T1ρ maps were comparable with 2D T1ρ maps for the depiction of cardiac structure such as left ventricle myocardium and papillary muscles, but 3D images presented visually less noise than 2D images. **(B)** Bull’s-eye plots of the myocardial T1ρ mean and CV measured by 3D and 2D sequences. The results in the 16 American Heart Association segments are averaged across all healthy volunteers. The CVs in the inferior-lateral segments were slightly higher for both sequences, potentially due to the attenuation of coil sensitivity in those regions. *CV* coefficient of variation, *2D* two-dimensional, *3D* three-dimensional

Short-axis T1ρ maps of three additional healthy subjects acquired both with 2D and 3D sequences are shown in Fig. 4**A**. The 3D T1ρ maps were visually comparable with the 2D T1ρ maps in depicting cardiac structures such as left ventricular myocardium and papillary muscles. Some differences between 2D and 3D images were observed due to the different readout sequences (bSSFP vs. SPGR). However, the 3D images reconstructed with HD-PROST exhibited reduced noise compared to the 2D images, which were acquired without denoising. Fig. 4**B** shows the bull's-eye plots of the myocardial T1ρ mean values and CVs measured with the 2D and the proposed 3D sequence averaged over all healthy volunteers. Both 2D and 3D sequences exhibit uniform T1ρ mean values across all segments, but the 3D sequence resulted in a slightly higher mean value (112.8 ± 16.7 vs. 106.1 ± 15.1%, p<0.01) and lower CV than the 2D sequence (10.2 ± 5.2 vs. 11.4 ± 4.4%, p = 0.02) comparing all 160 segments.

## 4. Discussion

In this study, we developed and validated a 3D free-breathing, high-resolution adiabatic T1ρ mapping sequence on a commercially available 0.55T MRI system. The proposed sequence demonstrated excellent accuracy as confirmed in phantom experiments. Notably, the use of a 3-parameter fitting model that incorporates both contrast-weighted images and a saturation-prepared image resulted in improved accuracy [11]. In addition, SAT-based sequence has better accuracy and robustness to heart rate variations as it resets the magnetization in every heartbeat and ensures a uniform signal baseline before T1ρ preparation. The scan efficiency is also improved without the necessity of idle heartbeats for magnetization recovery as in conventional 2D sequences. For in-vivo results, left ventricle T1ρ values measured with the conventional 2D and proposed 3D sequence show good agreement among all healthy subjects (**Figure S1**), while the 2D sequence exhibits a small negative bias compared with the 3D sequence (3.77 ms, 3%). This bias may be caused by the incomplete recovery of the longitudinal magnetization in the 2D sequence, thereby leading to a slight T1ρ underestimation.

A potential drawback of the proposed sequence is that the precision may be influenced by lower signal-to-noise ratio (SNR). SAT preparation pulses enhance both accuracy and heart rate robustness and have been widely used in previous parametric mapping sequences at 1.5T or 3T, although the SNR is decreased [3]. 3D acquisitions have higher SNR than 2D, thus can partially compensate for the signal loss and reduce the influence on precision [13]. At low-field strength, myocardial T1 at 0.55T (∼700 ms) is significantly shorter than that at 1.5T (∼1100 ms) or 3T (∼1300 ms) [2], thus, the longitudinal magnetization of the myocardium will recover more rapidly at 0.55T, which makes SAT preparation a good option at 0.55T given the lower SNR level compared to higher field strength systems. Another challenge is the smaller number of available receiver coil channels compared to higher field MR scanners, which can induce spatial variations of coil sensitivity and further reduce the SNR. To overcome the above limitations, the proposed 3D T1ρ mapping sequence employed patch-based low-rank regularization (HD-PROST) in concert with iterative SENSE image reconstruction, which can effectively reduce the noise in the weighted images [10], improve precision and maintain accuracy of the T1ρ maps (**Figure S2**). Consequently, the proposed sequence demonstrated good precision for whole-heart myocardial T1ρ assessment at 0.55T (CV: 10.2%).

It is also important to note that the absolute adiabatic T1ρ value is highly dependent on the specific parameters of the adiabatic-SL preparation pulse (i.e. $\tau_{\mathrm{HS}},\;B_{1,\max},\;\omega_{\mathrm{BW}},\;\mu \;$or β), as the nominal adiabatic T1ρ represents an average relaxation time throughout the adiabatic HS pulse. In addition, magnetic field strength influences MR relaxation times, including T1ρ. As a result, absolute T1ρ values measured with different SL parameters and/or at different field strengths may not be directly comparable. For example, a prior study performed at 1.5T using an HS pulse with $\tau_{\mathrm{HS}}$ = 20.5 ms, $B_{1,\max}$ = 13.5 µT (825 Hz), $\omega_{\mathrm{BW}}$ = 1500 Hz, and β = 6, reported a myocardial T1ρ value of approximately 138 ms [6]. Similarly, another study at 3T employed an HS pulse with $\tau_{\mathrm{HS}}$ = 30 ms, $B_{1,\max}$ = 13.5 µT, and validated different combinations of $\omega_{\mathrm{BW}}$ and β. Using $\omega_{\mathrm{BW}}$ = 900 Hz, and β= 6.9, the measured myocardial T1ρ was approximately 190 ms [5]. Since normal myocardial T1ρ values can vary substantially depending on sequence parameters and field strength, establishing local reference values in healthy subjects is essential before applying adiabatic T1ρ mapping in clinical settings.

## 5. Limitations

This study has several limitations. First, due to RF power limitations of the commercial low-field MRI system employed, the maximum achievable $\tau_{\mathrm{SL}}$ was restricted to 4$\tau_{\mathrm{HS}}$ (60 ms), which may have constrained the dynamic range, particularly for estimating longer T1ρ values. However, the use of a 3-parameter fitting model incorporating a SAT-prepared image helped partially mitigate this limitation [4]. As a result, the proposed sequence demonstrated reliable performance for myocardial T1ρ values around 110 ms. While extending $\tau_{\mathrm{SL}}$ could enhance the dynamic range, doing so would require upgraded RF hardware. Alternatively, applying 6 or 8 HS pulses with 10 ms gaps between HS pulses may be feasible, although T1 relaxation effects during these gaps could compromise T1ρ contrast.

Second, the acquisition sequence and reconstruction algorithm in this study may have potential limitations, making 3D images slightly blurrier and over-regularized compared to 2D images. For acquisition, reducing the number of k-space lines per readout or using a smaller readout flip angle could reduce the blurring effect from readout. However, it would either increase scan time or further reduce the SNR. For reconstruction, HD-PROST regularization with the current setting degraded the visual spatial resolution, although it improved the precision. The image quality may be improved by further optimizing the reconstruction methods. Finally, the proposed 3D T1ρ mapping technique requires further validation before it can be applied in patients. Large cohort studies are needed to evaluate its diagnostic utility and reproducibility.

## 6. Conclusion

The proposed method enabled 3D T1ρ mapping with 2 mm isotropic resolution in approximately 7 min and produced results comparable to breath-hold 2D T1ρ mapping, showing promising results for non-contrast, whole-heart myocardial tissue characterization at low-field strength.

## Funding

The authors acknowledge financial support from: (1) BHF programme grant RG/20/1/34802 and King’s BHF Centre for Award Excellence RE/24/130035, (2) Wellcome EPSRC Centre for Medical Engineering (NS/A000049/1), (3) Millennium Institute for Intelligent Healthcare Engineering ICN2021_004, ANID FONDECYT 1250261 and ANID FONDECYT 1250252, (4) IMPACT, Center of Interventional Medicine for Precision and Advanced Cellular Therapy FB210024 (5) the Department of Health through the National Institute for Health Research (NIHR) comprehensive Biomedical Research Centre award, (6) NIHR Cardiovascular MedTech Co-operative, and (7) the Technical University of Munich – Institute for Advanced Study. The views expressed are those of the authors and not necessarily those of the BHF, NHS, the NIHR or the Department of Health.

## Author contributions

**Dongyue Si:** Writing – review & editing, Writing – original draft, Methodology, Data curation, Conceptualization. **Michael G. Crabb:** Writing – review & editing, Data curation. **Simon J. Littlewood:** Writing – review & editing, Data curation. **Karl P. Kunze:** Writing – review & editing, Methodology. **Claudia Prieto:** Writing – review & editing, Supervision, Resources, Project administration, Funding acquisition. **René M. Botnar:** Writing – review & editing, Supervision, Resources, Funding acquisition, Conceptualization.

## Declaration of competing interests

The authors declare that they have no known competing financial interests or personal relationships that could have appeared to influence the work reported in this paper.
