# Supplementary material for: Free-breathing three-dimensional whole-heart adiabatic T1ρ mapping for non-contrast tissue characterization at 0.55T
Source: J Cardiovasc Magn Reson. 2025 Dec 24;28(1):102676. doi: 10.1016/j.jocmr.2025.102676 (PMC12814838; doi:10.1016/j.jocmr.2025.102676)
Supplement: Supplementary file 1 — Supplementary material [file mmc1.docx]

**METHODS**

***Data analysis***

For phantom experiments, the middle slices of the 3D T1ρ maps that have matched spatial position with the reference 2D T1ρ map were used for analysis. Circular regions of interest covering the center of each phantom vial were manually defined on each parametric map. T1ρ mean value, standard deviation (SD), and coefficient of variation (CV) (percentage of the SD divided by the mean) of the pixels inside ROI were calculated for each phantom vial. To assess the accuracy of the proposed 3D T1ρ mapping, linear regression was used to analyze the correlation and agreement of the measured T1ρ mean values in comparison with the reference values.

For analysis of the 3D T1ρ maps acquired in healthy subjects, multiple short-axis views were generated by double oblique reformatting of the 3D data matching the orientation and slice location of the 2D T1ρ mapping sequence. Approximately 40 short-axis slices with 2mm isotropic resolution were generated for each subject. The epicardial and endocardial contours of the left ventricle myocardium were manually segmented on each short-axis T1ρ map, and the segmented left ventricle was divided into 16 segments according to the American Heart Association (AHA) 17-segment model (excluding apical cap). The T1ρ mean values and CVs were used to evaluate the accuracy and precision, respectively, which were calculated in each AHA segment and the whole left ventricle for all healthy subjects. Bland-Altman analysis was performed to examine the agreements of the subject-wise T1ρ mean values obtained with the proposed 3D sequence and the 2D reference sequence. Paired two-tailed Student's t-test (α = 0.05) was used to analyze the statistical differences in segment-wise and subject-wise T1ρ mean values measured by the proposed and reference sequence. To display the spatial distribution of the T1ρ measurements across the whole left ventricle, T1ρ mean values and CVs in each AHA segment was averaged across all healthy subjects and visualized using bull's-eye plots.

**Table S1.** Imaging parameters of 2D and 3D T1ρ mapping sequences for phantom and in-vivo experiments. GRE, gradient echo; SPGR, spoiled gradient echo; bSSFP, balanced steady-state free precession; VD-CASPR, variable-density Cartesian trajectory with spiral-like profile order.

|  | **Phantom** | | **In-vivo** | |
| --- | --- | --- | --- | --- |
|  | **2D** | **3D** | **2D** | **3D** |
| **FOV (mm^2^/mm^3^)** | 126×360 | 320×320×40 | 306×360 | 320×320×~100 |
| **Resolution (mm^2^/mm^3^)** | 2.3×2.3 | 2×2×2 | 2.3×2.3 | 2×2×2 |
| **Slice thickness (mm)** | 10 | N/A | 10 | N/A |
| **Readout** | Single-echo GRE | SPGR | bSSFP | SPGR |
| **Acceleration** | no | 4×VD-CASPR | 2×GRAPPA | 4×VD-CASPR |
| **Flip angle (°)** | 90 | 25 | 90 | 25 |
| **TR/TE (ms)** | 10000/6.97 | 5.37/2.31 | 4.45/1.89 | 5.37/2.31 |
| **Bandwidth (Hz/pixel)** | 130 | 453 | 539 | 453 |
| **Lines per readout** | 1 | 28 | 45 | 20-30 |
| **Preparation pulses** | 0, 2HS, 4HS, SAT | SAT, SAT-2HS, SAT-4HS, SAT | 0, 2HS, 4HS, SAT | SAT, SAT-2HS, SAT-4HS, SAT |
| **Recovery time** | N/A | N/A | 3s | N/A |
| **Number of images** | 4 | | | |
| **Fitting model** | 3-parameter | | | |


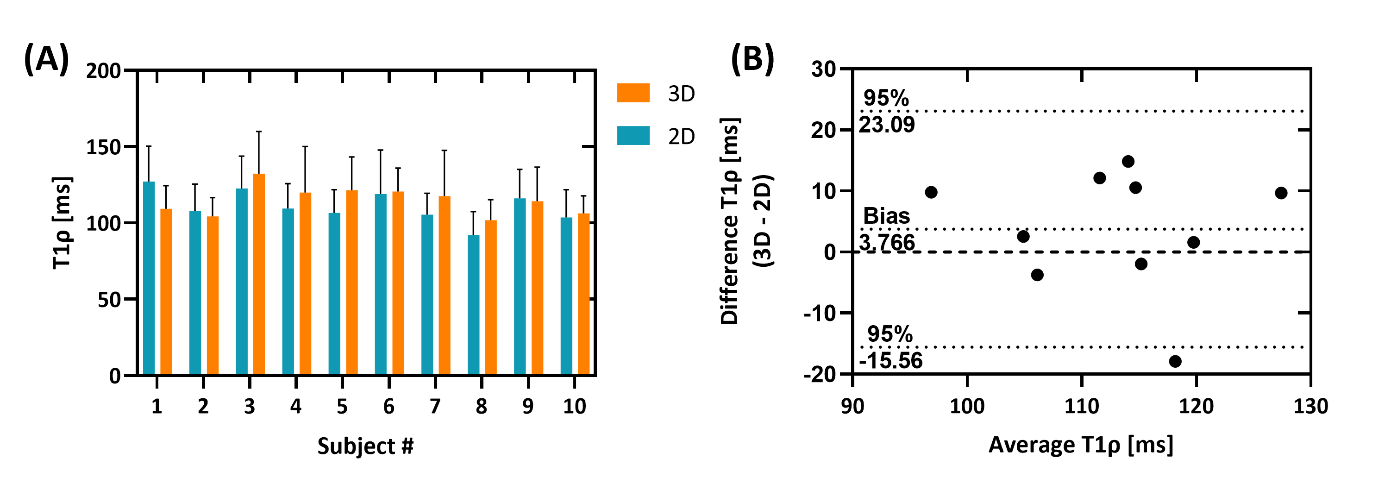


**Figure S1.** Statistical comparison of left ventricle T1ρ measured by the proposed 3D T1ρ mapping and breath-hold 2D T1ρ mapping. **(A)** Mean and standard deviation of all the 10 healthy volunteers measured by different sequences. **(B)** Bland-Altman analysis comparing mean T1ρ measured by 3D and 2D T1ρ mapping sequences. The results of the proposed 3D sequence were comparable with those of the 2D sequence (114.8 ± 9.4 vs. 111.0 ± 10.3 ms, p = 0.26 according to paired two-tailed Student's t-test) and exhibited a small bias of 3.7 ms.


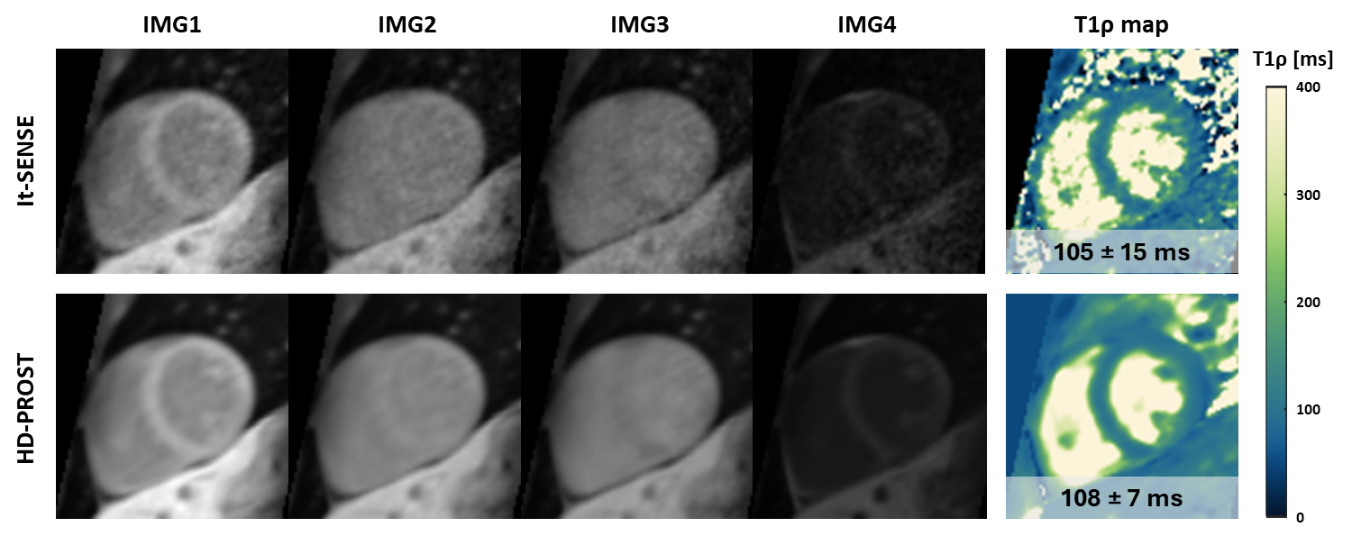


**Figure S2.** Representative contrast weighted images and T1ρ maps of a healthy subject obtained with the proposed 3D T1ρ mapping sequence using iterative SENSE (it-SENSE) reconstruction with and without high-dimensional patch-based low-rank regularization (HD-PROST). Septal myocardium T1ρ value is shown in each map respectively.
